# Phenotypic and Molecular Analyses of *Rhizoctonia* spp. Associated with Rice and Other Hosts

**DOI:** 10.3390/microorganisms7030088

**Published:** 2019-03-19

**Authors:** Regina Faye C. Sandoval, Christian Joseph R. Cumagun

**Affiliations:** 1 Institute of Weed Science, Entomology and Plant Pathology, College of Agriculture and Food Science, University of the Philippines Los Baños, College, Laguna 4031, Philippines; rcsandoval2@up.edu.ph; 2 Molecular Phytopathology and Mycotoxin Research, University of Göttingen, Grisebachstrasse 6, 37077 Göttingen, Germany

**Keywords:** *Rhizoctonia*, rice, molecular markers, phenotypic markers

## Abstract

Forty-two *Rhizoctonia* isolates were collected from rice, mung bean, and grasses from Laguna, Philippines. Sixteen isolates were binucleate *Rhizoctonia* (BNR), while 26 were multinucleate *Rhizoctonia* (MNR). BNR isolates produced white to brown, small sclerotia (<1.0 mm) except for mung bean isolates. Twenty MNR isolates produced big (>1.0 mm), light to dark brown sclerotia, three produced salmon-colored masses in the medium, and three did not produce sclerotia. Twenty-three MNR isolates were identified as *R. solani* AG1-IA using specific primers. Deduced Internal Transcribed Spacer (ITS) sequences of BNR isolates D1FL, NVL, and ScNL shared 100, 97, and 100% identity with *R. oryzae-sativae*, respectively, while MNR isolates BMgL, IbMgL, and MaSL that produced salmon-colored masses shared 100, 90, and 100% identity with *R. oryzae*, respectively. Preliminary analysis of the DNA fingerprint patterns generated by repetitive-element PCR (rep-PCR) clustered the 42 isolates into three: *R. solani*, *R. oryzae-sativae*, and *R. oryzae*, together with *Ceratobasidium* sp. *R. solani* isolates were pathogenic on rice (TN1), barnyard grass, mungbean (Pagasa 3), and tomato (Athena), while *R. oryzae* and *R. oryzae-sativae* isolates were only pathogenic on rice, *Echinochloa crus-galli*, and tomato. *R. solani* and *R. oryzae* were found to be more virulent than *R. oryzae-sativae*.

## 1. Introduction

Rice is a staple food in many countries across the globe including the Philippines. In 2009, it was recorded that Filipinos eat an average of 123 kg of rice per person annually—which was among the highest in the world [1]. About 4.7 million hectares is planted with rice in the country, producing about 18.9 million metric tons [2].

Among the major constraints in rice production are diseases. Rice sheath blight caused by *Rhizoctonia solani* was the second most important pathogen of rice in rice-growing countries for the last two decades [3]. *R. solani* produce lesions on the sheaths; however, under favorable conditions, these lesions can extend to the upper sheaths and to the leaves and even to the panicle. The infected sheaths/leaves will then dry out and die faster resulting in a reduction in canopy leaf area, thereby reducing yield [4]. *R. solani* is present wherever rice is grown and is responsible for at least 5% yield loss [5]. When susceptible cultivars were planted in United States, a yield loss as high as 50% was observed [4]. In Asia, annual yield losses of 10% and 20% due to sheath blight are observed in India and Thailand, respectively [5]. In the Philippines, Dilla observed 3.59 to 27.01% (0.34 to 1.09 t/ha) and 3.16 to 28.62% (0.35 to 1.66 t/ha) yield reduction during the wet and dry seasons, respectively [6].

There are two minor sheath pathogens of rice, *R. oryzae* and *R. oryzae-sativae*, which cause sheath spot and aggregate sheath spot, respectively. Both are considered minor diseases of rice; however, in a field study in Australia, results showed that aggregate sheath spot could cause yield losses up to 20%, while sheath spot can cause losses up to 10%. These diseases are becoming increasingly important in temperate rice-growing regions due to the widespread cultivation of susceptible semi-dwarf and high-yielding cultivars [7]. Both sclerotia of *R. oryzae* and *R. oryzae-sativae* insurvived better in plant residue than in the soil [8]. *Rhizoctonia* spp. are soil inhabitants which play various roles in the ecosystem as saprophytes, symbionts, and pathogens [9]. The genus exists primarily as sterile mycelium with varied forms and sizes of sclerotia and may either be uni-, bi-, or multinucleate [10]. *R. solani* is a complex species, wherein its isolates are further divided into anastomosis groups (AGs), based on the ability of the hyphae to fuse with the hyphae of a tester isolate. The complexity of *R. solani* does not stop at AGs because some AGs are further divided into subgroups based on cultural morphology, host range, virulence, and genetic characters [11].

Variability and diversity studies on *R. solani* associated with rice, as well as other economically important hosts, were conducted worldwide [12,13,14,15,16]. Using Inter Simple Sequence Repeat (ISSR) markers, large variation was found among rice-infecting isolates of *R. solani* AG1-IA in north India [17]. In Bangladesh, significant variation was observed in sclerotial size, shape, and distribution of 18 isolates of *R. solani* in rice [18]. In China, 43 haplotypes of *R. solani*, all pathogenic on five cultivars of rice, were identified based on Internal Transcribed Spacer (ITS) sequencing with high levels of diversity [19]. In the Philippines, the distribution of vegetatively compatible populations (VCPs) of *Rhizoctonia solani* AG1-IA in an experimental field planted with different host species at the Institute of Plant Breeding (IPB) at the University of the Philippines Los Baños (UPLB) was studied by Pascual and Hyakumachi [20]. A single VCP dominating the large experimental plot was observed, which does not conform to observations made in a neighboring Internartional Rice Research Institute (IRRI) experimental farm in Los Baños, as well as in other upland farms in Luzon, Philippines, where different VCPs were observed. Available resources on variability studies on *R. solani* in the Philippines are very limited, and this can be considered as one gap in the knowledge on Philippine *R. solani* population.

The variability in this pathogen complex increases the difficulty encountered by researchers in developing resistant host genotypes and deploying tolerant varieties [3]. Knowing the variability in a pathogen population will be helpful for researchers in breeding programs. Knowing the variability in the virulence pattern of the pathogen also helps in the evaluation and identification of resistant and susceptible genotypes. This study aimed to assess the variability in *Rhizoctonia* spp. causing sheath diseases in rice and its pathogenicity to other hosts. Specifically, it aimed to isolate *Rhizoctonia* spp. from rice and alternate hosts from irrigated lowland rice cropping systems in Laguna, Philippines; to characterize the isolates based on their colony morphology, as well as hyphae and sclerotial characteristics; to assess the genetic variability among the isolates using molecular techniques; and to evaluate the pathogenicity and assess the virulence pattern of *Rhizoctonia* spp. against rice and other hosts such as barnyard grass (alternate host), tomato, and mungbean. Tomato is a major host crop of the pathogen and mungbean is widely used as a rotation crop after rice in farmers’ fields in the Philippines.

## 2. Materials and Methods

### 2.1. Sample Collection and Isolation

Rice plants with sheath blight symptoms were randomly collected from the rice-producing areas in the southern part of Laguna province. Samples with at least two plants were collected from 59 barangays from 16 towns in Laguna and one town in Quezon. Weeds showing sheath blight symptoms were also collected and processed in the laboratory. Mungbean plants with dry stem rot were also collected from the experimental sites of National Crop Protection Center and Crop Science Cluster, both located at the University of the Philippines Los Baños, College, Laguna (UPLB). The samples were washed with running tap water to remove soil and other debris and blot dried. A 5 mm × 5 mm portion of the advancing region of the lesion (half healthy and half diseased) was cut and surface sterilized in 0.5% sodium hypochlorite for 1 min, followed by rinsing in sterile distilled water (sterile distilled H_2_O) three times. The cut leaf tissues were blot-dried in sterile filter paper or tissue and planted on water agar (WA). Hyphal tips with more or less perpendicular branching were transferred to potato dextrose agar (PDA) slants.

Isolates for preliminary identification were grown in PDA plates. Agar blocks of 48-h-old cultures were mounted in water to observe the mycelial and branching characteristics of the isolates. The following distinct morphological characters were observed: mycelia branched at acute to right angles, constrictions at the point of branching or near the point of branching, and septum formation near the branching point. The isolates were purified and maintained on PDA slants until further use.

### 2.2. Culture Characterization

Cultures were grown on PDA for 10 days and the following cultural characteristics were observed: colony color, growth pattern (abundant, moderate, or scarce), and sclerotial formation pattern (central, peripheral, or scattered). The diameter of sclerotia from 10-day-old cultures was also be measured (20 observations/isolate) using a ruler. Radial growth rate was also measured at 24, 48, and 72 h of incubation after inoculation. The characterization was conducted twice with three replicates per isolate per trial. Based on average colony growth diameters, the isolates were classified into fast (>65 mm), medium (60–65 mm), and slow (40–59 mm) growers, following the groupings by Lal et al. [15].

### 2.3. Microscopic Examination

The hyphal characteristics were also evaluated. The isolates were grown on a thin layer of PDA. An agar block (10 × 10 mm) was cut near the center of 48-h-old culture and rubbed lightly with a drop or two of wetting solution (1 mL of Tween 20 and 1 mL of 85% lactic acid in 1000 mL of dH_2_O). The mycelia of *Rhizoctonia* spp. were hydrophobic; thus, the agar blocks were first rubbed with wetting solution (WS) to be able to properly mount it in water. Excess wetting solution was removed by placing a tissue paper on the sides of the block and a drop or two of distilled water was added. The hyphal width was measured (20 observations/isolate) at 40× using the software DinoCapture 2.0 of the Dino-Eye eyepiece camera (AnMo Electronics Corporation, 17F, No. 97, Sec. 4, ChongHsin Rd., Sanchong Dist., New Taipei City, Taiwan). The formation of septa, and the constriction and angle of branching were also observed. Nuclear staining was done following the protocol of Herr [21]. An agar block (10 mm × 10 mm) with the hyphal tips of 48-h-old isolates on PDA was cut and rubbed with WS. Excess WS was removed and the agar blocks were stained with 0.5% aniline blue in lactophenol for 5 to 15 min. The hyphae were examined microscopically at 40× and the number of nuclei found in each cell was counted. Twenty cells were randomly selected from a location of strain gradient where nuclei and septa could be clearly observed. Aniline blue (0.5%) was used to stain 24-h-old hyphal tips and the number of nuclei present in each cell was counted [21].

### 2.4. DNA Extraction

The genomic DNA was extracted using the protocol by Cenis [22] with slight modification. A 1.5-mL microcentrifuge tube was filled with 500 μL of potato dextrose broth (PDB) and inoculated with hyphal threads of *Rhizoctonia* spp. The culture was allowed to grow for 72 h at 25 °C. The tubes were centrifuged for 5 min at 13,000 rpm to collect the mycelia, washed with 500 μL of Tris-ethylenediaminetetraacetic acid (EDTA) buffer and then pelleted again. The buffer was decanted and 300 μL of extraction buffer (200 mM Tris-HCl, pH 8.5, 250 mM NaCl, 25 mM EDTA, and 0.5% SDS) was added. The pelleted mycelium was crushed using a sealed 100–1000-μL micropipette tip (the tip was flamed for a few seconds until the micropipette tip melted and was sealed). Then, 150 μL of 3 M sodium acetate (pH 5.2) was added and tubes were placed at −20 °C for 10 min. Samples were centrifuged and the supernatant was transferred to a new tube. An equal volume of isopropanol (~450 μL) was added and allowed to stand at room temperature for at least 5 min. Precipitated DNA was collected by centrifugation at 13,000 rpm for 5 min. The DNA pellet was washed with 500 μL of 70% EtOH by flicking and was then centrifuged again to collect the DNA pellet. The alcohol was decanted, and the pellet was air-dried (approximately 30 min) and resuspended in Tris-EDTA buffer. The samples were kept at −20 °C until further use.

### 2.5. Detection of AG1-IA Isolates Using Specific Primers

A region of the 28S ribosomal DNA unit was amplified using primers specific for *R. solani* subgroup AG1-IA. Isolates belonging to the said subgroup produced amplicons of approximately 265 bp. Genomic DNA from test AG1-IA and AG1-IG isolates were used as positive and negative checks, respectively. The primer pairs *R. solani* AG-common primer (forward) AG-C 5′–CTCAAACAGGCATGCTC–3′ and *R. solani* subgroup-specific primer AG1-IA (reverse) 5′–CAGCAATAGTTGGTGGA–3′ were used for the PCR reaction as described by Matsumoto [23].

### 2.6. Amplification of ITS 1–5.8S–ITS 2 Regions

Fragments of the ribosomal DNA (rDNA) gene were amplified using primer pairs ITS 1 (5′ TCC GTA GGT GAA CCT GCG G 3′) and ITS 4 (5′ TCC TCC GCT TAT TGA TAT GC 3′) for forward and reverse reactions, respectively [24]. The 25-µL PCR cocktail mix was prepared with the following reagents: 12.5 µL of 2× *Taq* Master Mix (Vivantis), 0.5 µL each of forward and reverse primers (100 pmoL), 100 ng of genomic DNA, and nuclease-free water (Vivantis). The thermal cycler was programmed as follows: initial denaturation at 94 °C for 1 min; 94 °C for 15 s, 58 °C for 15 s, and 72 °C for 15 s for 30 cycles; 72 °C for 7 min for final elongation. The amplicons were resolved in 1% agarose gel and run at 100 V for 40 min. Amplicons of selected isolates were sent out to First BASE Laboratories Sdn Bhd (No. 7-1 to 7-3, Jalan SP 2/7, Taman Serdang Perdana, Seksyen 2, 43300 Seri Kembangan, Selangor, Malaysia) for sequencing services.

### 2.7. Analysis of ITS Sequences

Based on the chromatogram, noisy portions of the sequences were removed using the BioEdit sequence alignment editor. Edited sequences were submitted as a nucleotide query at the Basic Local Alignment Search Tool (BLAST) of the National Center for Biotechnology Information (NCBI).

### 2.8. DNA Fingerprinting of Rhizoctonia Isolates Using Repetitive-Element PCR (Rep-PCR)

Genetic variability among the isolates was analyzed using Rep-PCR [25], following the protocol used by Aye and Matsumoto [12]. Primers BOX repeat-based (BOXA1R) (5′–CTACGGCAAGGCGACGCTGACG–3′) were used separately for Rep-PCR analysis. A 25-µl PCR mix was prepared with 12.5 µL of 2× *Taq* Master Mix (Vivantis), 1.25 µL of primer (100 pmoL/µL), 3.5 µL of nuclease-free water (Vivantis), and 1.0 µL of genomic DNA (50 ng/µL). The thermal cycler was programmed as follows: initial denaturation at 95 °C for 7 min; 94 °C for 1 min, 52 °C for 1 min, and 72 °C for 8 min for 30 cycles; 72 °C for 16 min for final extension. Amplified products were resolved in 1% agarose gel and stained with Gel Red and viewed using an ultraviolet (UV) transilluminator.

Inconsistencies with the DNA fingerprint patterns were observed during the study. For BOXA1R, eight to 13 bands were observed during the first trial; however, when the samples were run for the second time, only two to five fragments were observed. For preliminary analysis, only the DNA fragments produced during the first trial for BOXA1R were analyzed. The patterns were scored based on the presence (1) or absence (0) of a band at a specific place. The scores were analyzed using PAST (Paleontological Statistics Version 3.11, Natural History Museum, University of Oslo) and a dendrogram were generated from the cluster analysis of the results, generated by similarity coefficients using the unweighted pair group arithmetic average (UPGMA) analysis.

### 2.9. Pathogenicity Test

**Rice:** Representative isolates that were used for pathogenicity and virulence studies were selected based on growth rate and size of sclerotia. Mycelia discs (10 mm in diameter) were excised from three-day-old mycelia culture grown on PDA. Pathogenicity and virulence evaluation on rice was done using the micro-chamber screening method developed by Jia et al. [26]. The susceptible (TN1) rice variety was used. Rice seeds were soaked in water for 48 h and transferred to seedling pans. Seven-day-old seedlings were transferred to sealed clay pots. Three seedlings were planted at least 3 cm apart per pot with three pots (replicates). The seedlings were inoculated during the V3 stage or at approximately 20 days after emergence. The experiment was conducted three times.

Following the rapid micro-chamber screening method developed by Jia et al. [26], mycelial discs were placed and pressed to the base of the stem and wrapped with Parafilm to ensure that the mycelium was in contact with the plant. A PDA disc was used as negative control. Each pot was covered with a soft-drink bottle (clear or green) from which the bottom and cap were removed. The bottle was pushed down into the soil to create a micro-humidity chamber. The pots were arranged in a completely randomized design. During the days where temperature was forecasted to exceed 38 °C, the bottles were removed before noon and returned before sunset. Seedlings were cut at the ground line after 10 days, and lesion length was measured, as well as the length of the culm. The relative lesion height (RLH) was calculated by dividing the lesion height by the plant height and multiplying by 100. The RLHs were tested for the presence of outliers, normality (Shapiro–Wilk), and homoscedaticity (Levene’s). Only trials 1 and 2 passed the three tests (assumptions for ANOVA); thus, the data were combined, and one-way ANOVA was carried out using SPSS.

**Mungbean:** Mungbean seeds var. Pag-asa 3 (Institute of Plant Breeding, UPLB) were allowed to germinate on moist filter paper in petri dishes at room temperature for two days. Seedlings with similar germination rate were transferred to 15-cm-diameter plastic pots. Two seedlings were planted per pot with three pots (replicates) per trial. Ten-day-old seedlings were inoculated following the same procedure used in rice. The experiment was conducted twice. The same parameters and statistical analysis as in rice were employed.

**Tomato:** A true expanded leaf of 21-day-old tomato seedlings var. Athena (Condor Hybrid Seeds) were inoculated with filter paper discs (previously grown with mycelia *Rhizoctonia* spp.). The mycelial filter paper discs were stapled onto the leaf, and plants were again covered with soft drink bottles to create a micro humidity chamber. The inoculated plants were incubated for five days and disease severity (DI) was scored using an ordinal scale of 0–5, following the protocol by Castroagudin [27]. The scale represents a range of percentages of plant area affected (aa) with necrosis and/or chlorosis, where 0 = no infection, 1 = >0.0 to 15% aa with chlorosis and/or necrosis, 2 = >15 to 30% aa with chlorosis or necrosis, 3 = >30 to 60% aa with chlorosis or necrosis, 4 = >60 to 90% aa with chlorosis or necrosis, and 5 = > 90 to 100% aa with chlorosis or necrosis or dead plant.

The ordinal values were transformed into a continuous variable using the midpoint method and scoring scale by Castroagudin [27]. The following scale was used for scoring disease severity (DI) with corresponding midpoint (mp) value: 0 = 0% aa (no infection), mp = 0.0; 1 = >0.0 to 15.0% aa, mp = 8.0; 2 = >15.1 to 30.0% aa, mp = 23.0; 3 = >30.1 to 60.0% aa, mp = 45.5; 4 = >60.1 to 90% aa, mp = 75.5; 5 = >90.1 to 100% aa or dead plant, mp = 95.5. The same statistical analysis as in rice and mungbean was employed.

**Barnyard grass:** The pathogenicity of selected *Rhizoctonia* isolates was also tested on *Echinochloa crus-galli*, a common and widespread weed in paddy rice fields. Field-collected panicles were sun-dried, and seeds were transferred to sealed germinating pans. Very low germination rate was observed; thus, another procedure was tried. The sun-dried seeds were placed in moist filter paper just like in mungbean; however, again, very low germination rate was observed. When the leftover seeds were again submerged in water in sealed germinating pans, high germination rate was finally observed. It was found out that *E. crus-galli* seeds undergo a dormancy period of 3–4 months. Unfortunately, due to a lack of seeds, only one trial was conducted. Twenty-day-old seedlings were inoculated with mycelial discs using the same procedure in rice, with two seedlings per pot and three pots (replicates) per treatment. The same parameters and statistical analysis as in rice were employed. The isolates failed the Levene’s test; thus, the robust tests for equality of means, Welch and Brown–Forsythe, were carried out. Since the comparison of means between isolates was significantly different (*p* = 0.000) for both tests, as well as ANOVA, post hoc analysis was carried out with Scheffe’s test.

## 3. Results and Discussion

### 3.1. Collection and Isolation

**Figure 1:**
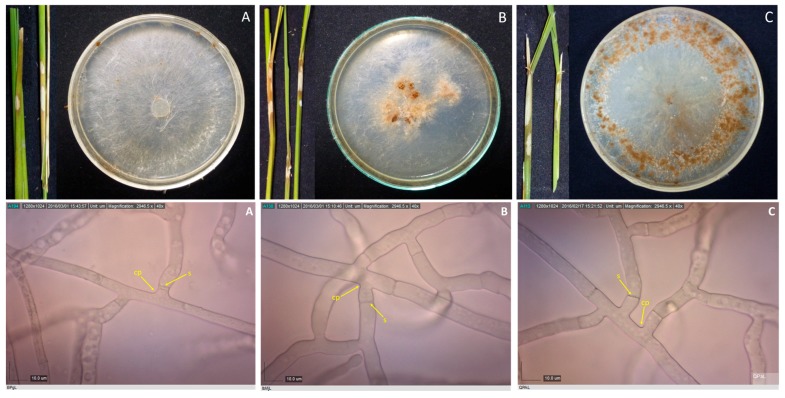
Rice stems showing sheath blight symptoms, and 10-day-old cultures of their corresponding pathogens, as well as their photomicrographs showing the mycelia branching at an acute to right angle with slight constriction at the point (cp) of branching and septum (s) formed near the branching point for isolates BPgL (**A**), IbMjL (**B**), and QPaL (**C**). Samples were viewed at 40×.

**Figure 2:**
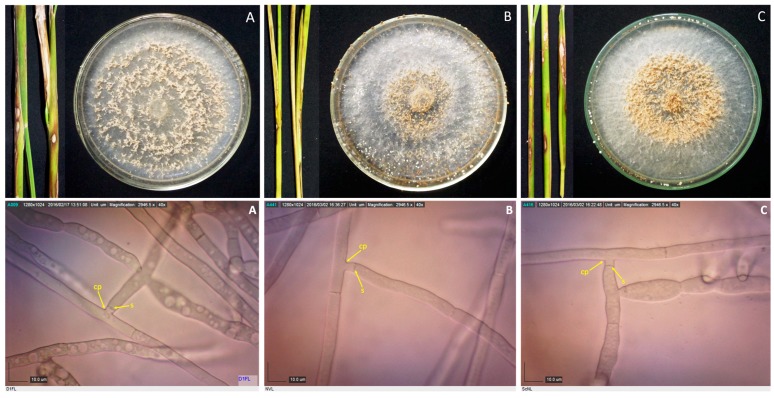
Rice stems showing sheath blight symptoms, and 10-day-old cultures of their corresponding pathogens, as well as their photomicrographs showing the mycelia branching at an acute to right angle with slight constriction at the point (cp) of branching and septum (s) formed near the branching point for isolates D1FL (**A**), NVL (**B**), and ScNL (**C**). Samples were viewed at 40×.

**Figure 3:**
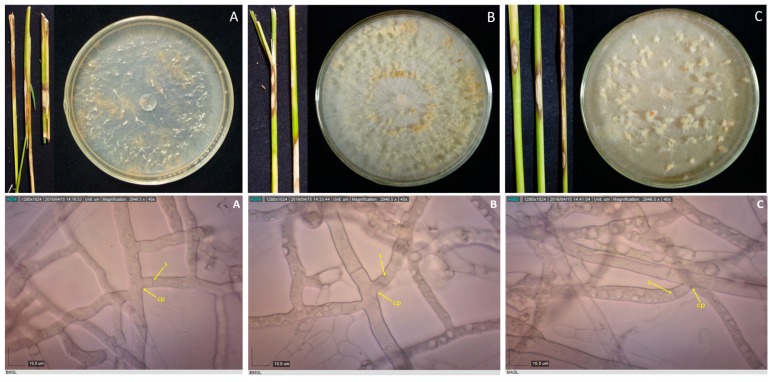
Rice stems showing sheath blight symptoms, and 10-day-old cultures of their corresponding pathogens, as well as their photomicrographs showing the mycelia branching at an acute to right angle with slight constriction at the point (cp) of branching and septum (s) formed near the branching point for isolates BMgL (**A**), IbMgL (**B**), and MaSL (**C**). Samples were viewed at 40×.

**Figure 4:**
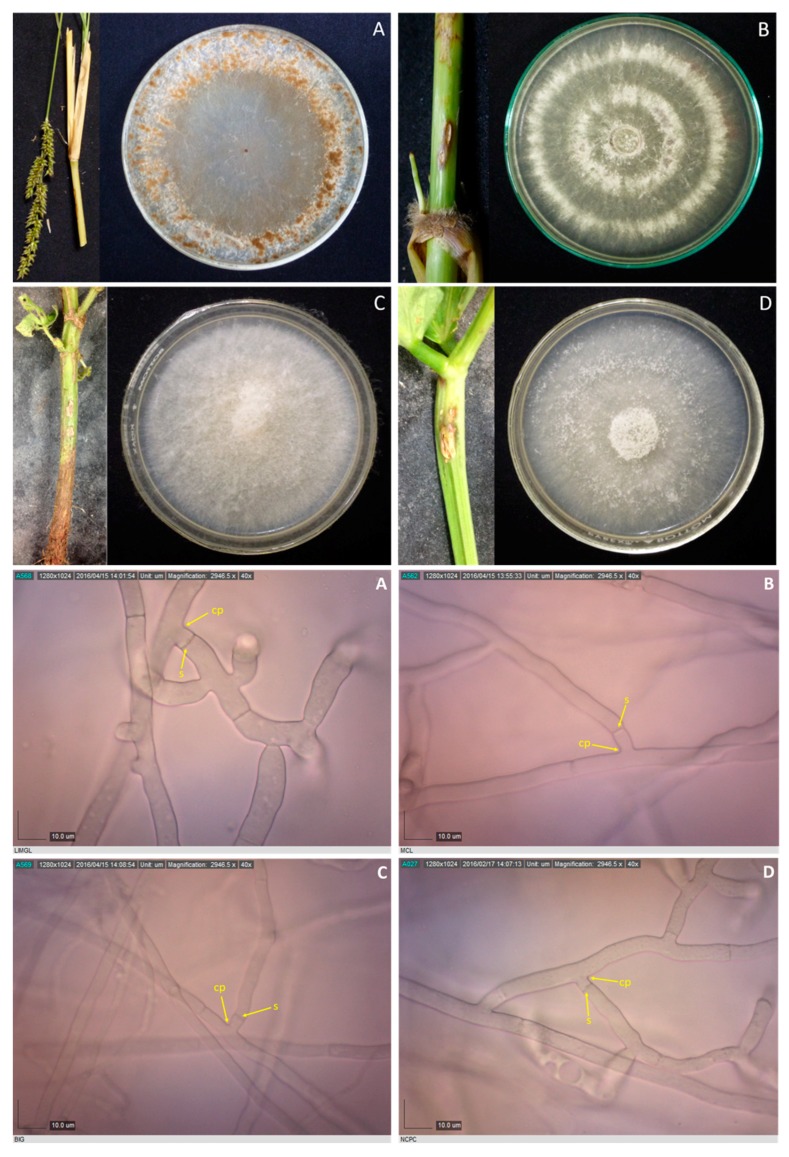
*Rhizoctonia* spp. isolated from *Echinochloa crus-galli* LiMgL (**A**) and unidentified grass MCL (**B**), and mungbean BIG and NCPC (**C** and **D**), and their corresponding photomicrographs showing mycelia branching at an acute to right angle with slight constriction at the point (cp) of branching and septum formed near the branching point (s). Samples were viewed at 40×.

Samples with sheath blight symptoms were collected from 59 barangays from 16 towns in Laguna and one town in Quezon. Forty-two *Rhizoctonia* spp. were isolated from rice, mungbean, and two grasses (Table 1). Duggar [28] described the concept of species of *R. solani* with young hyphae branching at an acute to right angle, with slight constriction at the point of branching, and septum formed near the constriction point [29]. These three characteristics were observed in all the isolates; thus, they were initially identified as *Rhizoctonia* spp. (Figure 1, Figure 2, Figure 3 and Figure 4).

### 3.2. Culture Characterization

Among the 42 isolates, 29 had fast mycelial development, 11 had medium development, and two showed slow mycelial development (Table 1). Most of the isolates produced light-brown cultures with moderate mycelial growth. Only four (ALQ, LiMgL, BPL, and SPL) isolates were observed to have abundant mycelial growth while three (BMgL, SfVL, and D1FL) had scarce mycelial growth. One (SfVL) isolate produced a whitish-brown culture, while five (IbMgL, MCL, MaSL, BIG, and NCPC) were yellowish brown, one (SPL) was brown, and one (BMgL) was light salmon (Table 1). *R. solani* isolates are known as fast growers that produce hyaline mycelia when young, which eventually turns yellow to brown with age (Gnanamanickam 2009).

### 3.3. Sclerotia Characterization

**Figure 5:**
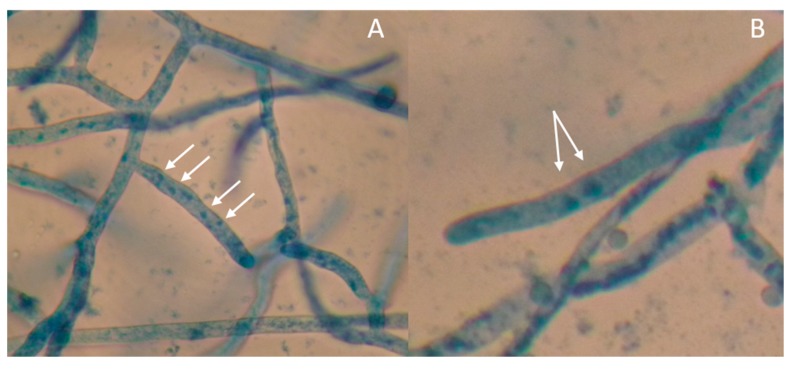
Photomicrograph of 24-h-old hyphal tips stained with 0.5% aniline blue showing multinucleate *Rhizoctonia* isolate LScL (**A**) and binucleate *Rhizoctonia* isolate BIG (**B**) as indicated by the arrows. Samples were viewed at 40×.

Sixteen isolates (38%) were observed to be binucleate *Rhizoctonia*, while 26 (62 %) were multinucleate (Figure 5, Table 2). The binucleate *Rhizoctonia* produced white to brown, small sclerotial bodies (<1.0 mm) but in excellent (>70 sclerotia/plate) number, except for mungbean isolates BIG and NCPC, which did not produce sclerotial bodies. Among the 25 multinucleate *Rhizoctonia*, 20 produced big (>1 mm) light-brown to dark-brown sclerotial bodies in fair (1–10) to very good (41–60) amounts, three produced salmon-colored masses that were embedded in the growth medium (BMgL, IbMgL, and MaSL), and three did not produce sclerotial bodies (LScL, PScL, and TaVL).

*R. solani* isolates, specifically those belonging to subgroup AG1-IA, produced brown to dark-brown sclerotia that were relatively spherical and large (1–3 mm) [30]. *R. oryzae-sativae* produced globose, white to brown sclerotia with a diameter of 0.5 to 2 mm, while *R. oryzae* produced salmon-colored sclerotia on formless masses that were waxy, soft, and usually embedded in the agar [7].

The sclerotial bodies were formed in central, peripheral, scattered, or ring patterns. Twenty-eight of the isolates had sclerotia scattered all over the plates. Five had sclerotia formed at the center. The peripheral formation pattern was observed for three isolates, and two isolates had a ring pattern. The hyphal width of 48-h-old cultures was measured using DinoCapture 2.0. Agar blocks were mounted in distilled water and 20 observations were recorded per isolate. The average hyphal widths of the isolates ranged from 4.60 to 9.43 µm. Hyphal widths of *R. solani*, *R. oryzae*, and *R. oryzae-sativae* generally range from 8–12 µm [31], 6–10 µm, and 3.5–7.0 µm [7], respectively. Thirty-eight *Rhizoctonia* spp. were isolated from rice, wherein three were suspected as *R. oryzae* (teleomorph: *Waitea circinata*) (8%), 13 (34%) as *R. oryzae-sativae* (teleomorph: *Ceratorhiza oryzae-sativae*), and 22 (58%) as *R. solani* (teleomorph: *Thanatephorus cucumeris*), based on cultural and microscopic characteristics. Three representative isolates each from the suspected *R. solani* (BPgL, IbMjL, and QPaL) and *R. oryzae-sativae* (D1FL, NVL, and ScNL) isolates were chosen for pathogenicity and virulence determination on rice and other hosts. The three suspected *R. oryzae* (BMgL, IbMgL, and MaSL) isolates, along with the non-rice *Rhizoctonia* spp. isolates (BIG, NCPC, LiMgL, and MCL), were also further characterized molecularly and phenotypically.

### 3.4. Direct Detection Using Specific Primers

**Figure 6:**
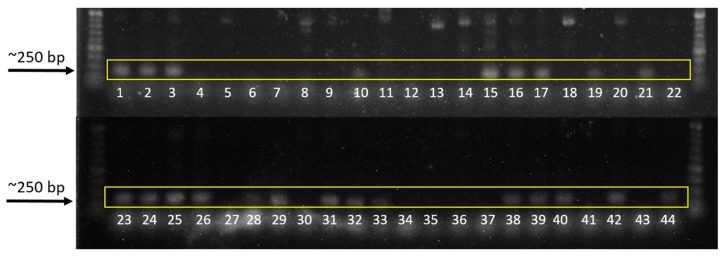
Gel documentation of the resolved DNA products using the specific primers for *R. solani* AG1-IA. Isolates belonging to subgroup AG1-IA are expected to produce a band of ~265 bp. Test cultures of AG1-IA (lane 21) and AG1-IG (lane 22) were used as positive and negative checks, respectively. Suspected *R. solani* isolates produced a band size of ~250 bp (lanes 1–3, 10, 15–17, 19, 21, 23–26, 28–29, 31–33, 38–40, 42, and 44). The suspected *R. oryzae-sativae* isolates (lanes 4–6, 14, 18, 20, 27, 30, 34–37, 41, and 43) and *R. oryzae* (lanes 7–9) did not produce 250-bp bands. Mungbean isolates BIG and NCPC (lanes 12 and 13) did not produce 250-bp bands.

Isolates belonging to the subgroup AG1-IA are expected to produce a 265-bp band. Test cultures of AG1-IA and AG1-IG were used as positive and negative checks, respectively. The positive check AG1-IA produced a ~250-bp band (Figure 6, lane 21), while the negative check AG1-IG did not produce any band (Figure 6, lane 22).

Among the 42 isolates, 23 (55%) were identified as *R. solani* AG1-IA as they produced a band size of ~250 bp, similar to that produced by the positive check (AG1-IA). These isolates were multinucleate and they produced large, brown sclerotial bodies. Bands of ~250 bp were not observed for binucleate *Rhizoctonia* and presumed *R. oryzae* isolates.

Pairings of anastomosis groups (AG) are usually used to identify the subgroup of *R. solani* isolates; however, the technique is tedious and requires a lot of practice. This direct detection method developed by Matsumoto [23] was found to be a helpful tool in the identification of *R. solani* AG1-IA isolates in this study. *R. solani* AG1-IA, the causal organism of rice sheath blight, is the most common among the *Rhizoctonia* AGs and is considered a significant pathogen that causes major economic impact in many major rice-growing countries [12]; thus, correct and rapid identification of the pathogen is important.

### 3.5. Amplification of ITS 1–5.8S–ITS 2 Regions

**Figure 7:**
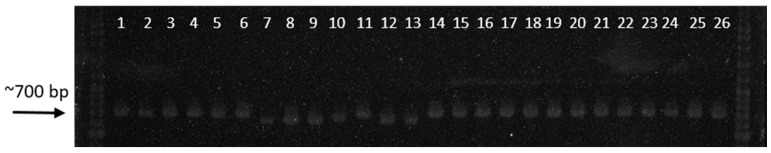
Digital image of ITS products of different *Rhizoctonia* spp. showing bands of approximately 650–700 bp. Suspected *R. solani* isolates produced a single band of ~700 bp, while the suspected *R. oryzae* and three binucleate *Rhizoctonia* isolated from other hosts produced a single band of ~650 bp.

Thirty-six isolates produced a band size of approximately 700 bp (Figure 7). The deduced ITS sequences (Table 3) of the representative isolates from binucleate *Rhizoctonia* (D1FL, NVL, and ScNL) shared 97–100% identity with *Ceratorhiza oryzae-sativae* (anamorph: *Rhizoctonia oryzae-sativae*). Isolate LiMgL from *E. crus-galli* also produced a 700-bp band and shared 97% identity with *R. solani.* A representative sample from the detected *R. solani* AG1-IA isolates (IbMjL) shared 99% identity with *R. solani f. sp. sasakii.* The remaining six isolates produced a band size of approximately 650 bp (Figure 7, lanes 7–10). Based on the deduced ITS sequences (Table 3), isolates BMgL and MaSL shared 100% identity with *Waitea circinata* (anamorph: *Rhizoctonia oryzae*), while IbMgL shared only 90% similarity with *W. circinata*. Isolates MCL, BIG, and NCPC shared 98, 99, and 99% identity with *Ceratobasidium* sp. AG-Ba, AG-L, and AG-A, respectively.

### 3.6. Rep-PCR

**Figure 8:**
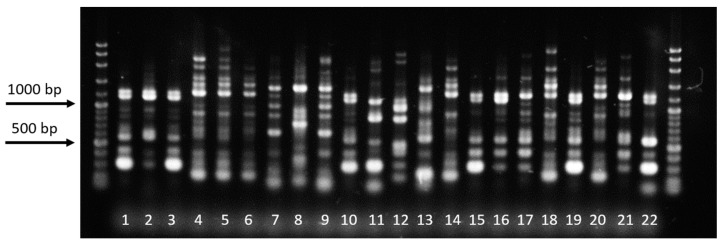
Examples of fingerprint patterns generated by BOXA1R primer. Lanes 1–3 and 10, 4–6, 7–9, and 11–13 show the fingerprint patterns produced from *Rhizoctonia solani*, *R. oryzae-sativae*, *R. oryzae*, and *Ceratobasidium* sp., respectively.

**Figure 9:**
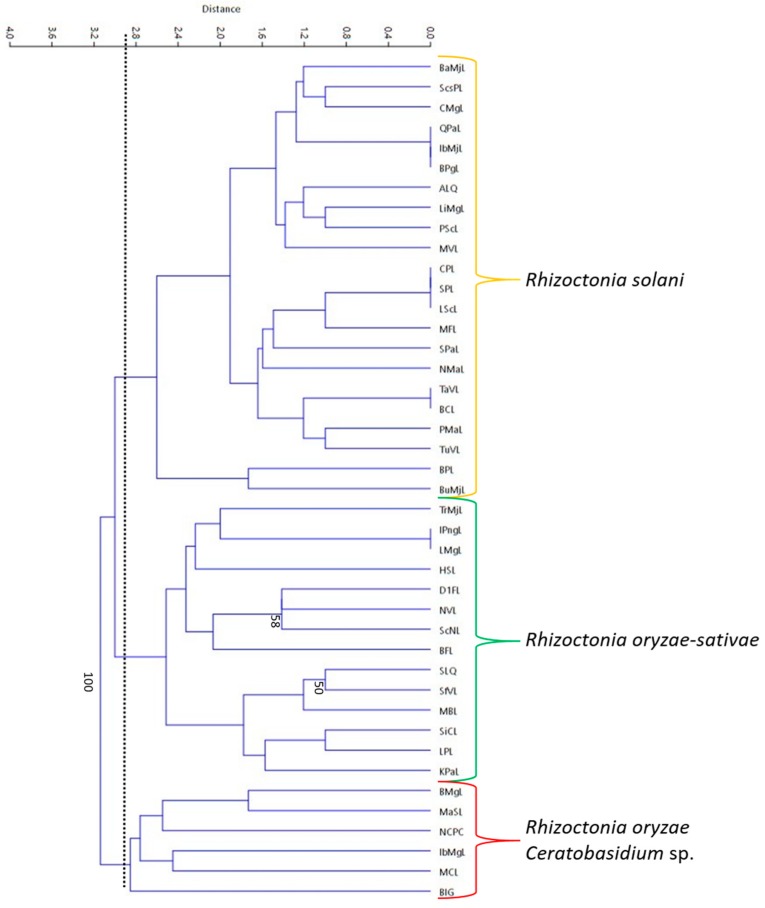
The repetitive-element PCR (Rep-PCR) generated dendrogram for the 42 *Rhizoctonia* isolates. A 2.9 similarity index (as measured by distance) was used for the interpretation of relatedness as depicted by the vertical line. Isolates clustered into three groups: *R. solani*, *R. oryzae-sativae*, and *R. oryzae* and *Ceratobasidium* sp. Bootstrap values below 50 were no longer included in the dendrogram.

Repetitive-element polymerase chain reaction (Rep-PCR) was conducted using BOXA1R primer and a dendrogram was generated from the fingerprint patterns (8–13 bands) produced by the 42 isolates (Figure 8). Based on the dendrogram, the isolates were clustered into three groups (at 2.9 similarity index) with relatively low bootstrap values. Identified *R. solani* AG1-IA isolates were clustered into one group, while the presumed *R. oryzae-sativae* isolates were also grouped together (Figure 9). The *R. oryzae* isolates were clustered together with the *Ceratobasidium* sp. It can be observed from the dendrogram that most of the closely related isolates do not belong to the same municipality, just like isolates BPgL, IbMjL, and QPaL, which were collected from Pagsanjan, Majayjay, and Paete, Laguna, respectively.

### 3.7. Pathogenicity Test

**Rice:** Among the nine rice and one grass isolates tested, *R. solani* isolate LiMgL from barnyard grass was the most virulent with a mean relative lesion height (RLH) of 51.41%, which was significantly higher than the RLHs of all the other isolates tested (Table 4). The RLH of *R. solani* isolates BPgL, IbMjL, and QPaL, as well as those of *R. oryzae* isolates BMgL, IbMgL, and MaSL, were not significantly different from each other. The RLHs of *Ceratorhiza oryzae-sativae* isolates D1FL, NVL, and ScNL were significantly lower than those of *R. solani* and *R. oryzae* isolates.

*R. solani* causes sheath blight of rice, which is considered as a major constraint to rice production [3], while *R. oryzae* and *R. oryzae-sativae* causes sheath spot and aggregate sheath spot of rice, respectively, which are only considered as minor diseases of rice. *R. oryzae* isolates were found to be equally virulent with *R. solani* rice isolates based on the observed RLHs. In the Philippines, Shahjahan et al. [32] observed *R. oryzae* in 10% of the rice fields they surveyed. The *R. oryzae-sativae* isolates were the least virulent; however, since they comprised 38% of the rice isolates randomly collected in this study, it does not make them a less important group of *Rhizoctonia* pathogens. Based on our knowledge, this is the first report of *R. oryzae-sativae* in the Philippines. The other binucleate *Rhizoctonia* (*Ceratobasidium* sp. AG-Ba, AG-L, and AG-A) were also tested for pathogenicity on the susceptible variety, TN1. Isolates BIG, MCL, and NCPC can also infect rice but were only able to produce very small lesions in some of the test plants with mean RLHs of 4.39%, 5.86%, and 4.38%, respectively. The RLHs of the three isolates were not included in the ANOVA since they failed the test for normality. The 13 *Rhizoctonia* isolates used in the experiment were successfully re-isolated from the test plant, thus confirming their pathogenicity on rice.

A Pearson product–moment correlation analysis was run to determine the relationship between growth rate and relative lesion height. The data did not violate tests for normality, linearity, and homoscedasticity. Results showed that there was a strong positive correlation between growth rate and pathogen virulence (as evaluated by relative lesion height), which was very highly significant (*r* = 0.817, *n* = 10, *p* < 0.0005).

**Barngrass:** Pathogenicity and virulence of the 13 selected *Rhizoctonia* isolates were evaluated using the micro chamber method used in rice. Sheath blight symptoms were observed in plants inoculated with all the isolates except for BIG. The pathogenicity of the 12 isolates was confirmed by re-isolating the *Rhizoctonia* pathogen from the test plants. The RLHs were again tested for the presence of outliers, normality, and homoscedasticity. Since all the 12 pathogenic isolates failed Levene’s test for homogeneity of variance, the Welch and Brown–Forsythe robust tests of equality of means were carried out. Since the comparison of means between isolates was significantly different (*p* = 0.000) for both robust tests, as well as for ANOVA, post hoc analysis was carried out with Scheffe’s test.

Isolates BPgL and QPaL were the most virulent against barnyard grass with mean RLHs of 58.78% and 58.08%, respectively. The RLHs of BPgL and QPaL were not significantly different from those of IbMjL, BMgL, IbMgL, MaSL, and LiMgL, but were significantly higher than those of binucleate *Rhizoctonia*. The results show the ability of *E. crus-galli* to serve as a good alternate host for *Rhizoctonia* spp. during the fallow period or when fields are planted with tolerant to moderately resistant rice varieties. *E. crus-galli* is included in the global compendium of weeds and is considered as the world’s worst weed in rice paddies [33]. In Laguna, Philippines, Furoc et al. [34] were able to collect 3.88 to 11.07 t/ha of grasses and broadleaf weeds with *E. crus-galli* being the dominant species.

Sheath blight is one of the most widely spreading diseases in paddy fields, as its causal pathogen, *R. solani*, not only infects rice, but also other paddy weeds like *Arachis hypogea*, *Cyperus iria*, *E. colonum*, and *E. crus-galli*, among others. The presence of sclerotial inoculum in soil, as well as in infected weeds in and around the fields, may help in spreading the disease, since symptoms in rice are usually observed near the bunds of rice fields where the infected weeds were growing [35]. Very low germination rates (only five out of 200) were encountered during the first attempts in this study. During the third attempt to set up the experiment, high germination rates were observed, but germination was not synchronized; thus, it was difficult to get enough plants of the same height to be used in a single trial. Due to limited availability of seeds, only one trial was conducted. *E. crus-galli* seeds can easily be collected from rice fields during the milking to harvesting stage, but *E. crus-galli* undergoes a dormancy period of three to four months [33], explaining the very low germination rates experienced during the first attempts.

Pearson product–moment correlation analysis was run to determine the relationship between the virulence level of selected *Rhizoctonia* isolates on rice variety TN1 and *E. crus-galli*. A strong, positive correlation was observed between the virulence levels on rice variety TN1 and *E. crus-galli*, which was very highly significant (*r* = 0.889, *n* = 10, *p* < 0.0005).

**Mungbean:** Only the *R. solani* isolates BPgL, IbMjL, QPaL, and LiMgL, and the mungbean isolates BIG and NCPC were pathogenic on mungbean. *R. oryzae*, *R. oryzae-sativae*, and *Ceratobasium* sp. isolates did not produce any lesions on the plant. The RLHs for mungbean were not normally distributed; thus, a Kruskal–Wallis H Test was used instead of one-way ANOVA. Results of the test showed that there was no significant difference in the RLHs among the different isolates (*x*^2^(2) = 0.261, *p* = 0.967) with a mean rank score of 24.08 for BPgL, 25. 42 for IbMjL, 23.00 for QPaL, and 25.50 for LiMgL. Only the RLHs for *R. solani* isolates were used in the analysis, because, out of the 12 plants inoculated with BIG for the two trials conducted, only three plants produced lesions, while, in those inoculated with NCPC, only one was observed with blighting on the stem. However, pathogenicity of BIG and NCPC, and the other four isolates was confirmed, since the pathogens were successfully re-isolated from the test plants.

Mungbean is widely grown in southeast Asia, Africa, South America, and Australia [36]. In the Philippines, it is recommended to grow mungbean after rice because it presents a good opportunity for farmers to earn additional income as the agricultural input needed by mungbean production is minimal [37]. Fortunately, the *R. oryzae* and *R. oryzae-sativae* isolates were found to be non-pathogenic on mungbean, but the *R. solani* isolates were pathogenic. Mungbean may also serve as a relay host for *R. solani* from rice since it is a soil-borne pathogen and infected rice stubbles are just left in the field. Butranu [38] evaluated the carrying capacity of component crops (rice, mungbean, and corn) of *R. solani* inoculum in relation to multiple cropping in field experiments conducted at the International Rice Research Institute (IRRI) in Los Baños, Laguna. Higher sheath blight intensity was observed for the mungbean–rice–mungbean cropping pattern with minimal cultural practices than for the corn–rice–mungbean cropping pattern, because the mungbean–rice–mungbean cropping pattern had higher carrying capacity of viable sclerotia than the corn–rice–mungbean cropping pattern.

**Tomato:** All the inoculated *Rhizoctonia* isolates were found to be pathogenic on tomato using the micro chamber humidity set-up. The rating used was an ordinal scale; thus, the scores were transformed to a continuous variable using the midpoint method. The transformed scores were tested for the presence of outliers, normality, and homoscedasticity. The scores were not normally distributed; thus, instead of one-way ANOVA, the Kruskal–Wallis H Test was used. Results showed that there were significant differences in the disease indices among the different isolates (*x*^2^(2) = 59.091, *p* = 0.000), with a mean rank score of 20.50 for isolates LiMgL, D1FL, NVL, and ScNL, 26.00 for BMgL, 31.50 for MaSL, 37.00 for NCPC and MCL, 48.00 for IbMgL, 53.50 for BIG, 62.75 for IbMjL and QPaL, and 73.00 for BPgL (Table 4). *R. solani* and *R. oryzae* isolates were observed to be more virulent on tomato than *R. oryzae-sativae* isolates.

During the collection trips for this study, it was observed that tomato was also used as an alternate crop, along with string beans, radish, pechay, eggplant, and squash, to rice. The practice of alternating rice and vegetables was widely observed in Majayjay, Laguna, where vegetable production is quite favorable, since it is elevated compared to other rice-producing towns in Laguna. Since the *Rhizoctonia* isolates were also pathogenic on tomato, they can cause a problem in its production when conditions become favorable for disease development.

For the future outlook of this project, it would be worthwhile to test additional AG-1-IA isolates belonging to different genetic backgrounds in order to obtain a comprehensive picture of the pathogenicity of the isolates.

## Figures and Tables

**Table 1:** Cultural characteristics of *Rhizoctonia* spp. on potato dextrose agar (PDA) medium. N—north; E—east; W—west; UPLB—University of the Philippines Los Baños.

#	Isolate	Host	Plant Part	Place of Collection	GPS Coordinates	Colony Color ^a^	Growth Pattern ^b^	Colony Growth Diameter (mm) ^c^			Mean Diameter (mm)
								24 h	48 h	72 h	
1	ALQ	Rice	sheath	Ayuti, Lucban, Quezon	14.10876° N 121.537007° E	light brown	abundant	51.67	90.00	90.00	77.22
2	SLQ	Rice	sheath	Samil, Lucban, Quezon	14.109835° N 121.525667° E	light brown	moderate	32.00	61.83	90.00	61.28
3	BaMjL	Rice	sheath	Bakya, Majayjay	14.16061° N 121.490699° E	light brown	moderate	61.83	90.00	90.00	80.61
4	BuMjL	Rice	sheath	Buharaw, Majayjay	14.114552° N 121.50457° E	light brown	moderate	50.33	90.00	90.00	76.78
5	IbMjL	Rice	sheath	Ilayang Banga, Majayjay	14.15° N 121.48° E	light brown	moderate	61.17	74.33	74.67	70.06
6	TrMjL	Rice	sheath	Talortor, Majayjay	14.15° N 121.46° E	light brown	moderate	33.33	69.33	90.00	64.22
7	BMgL	Rice	sheath	Burlungan, Magdalena	14.18434° N 121.442995° E	light salmon	scarce	40.33	85.17	90.00	71.83
8	CMgL	Rice	sheath	Cigapas, Magdalena	14.22821° N 121.435905° E	light brown	moderate	56.33	87.83	90.00	78.06
9	IbMgL	Rice	sheath	Ibabang Bugtong, Magdalena	10.178268° N −74.495031° W	yellowish brown	moderate	54.67	90.00	90.00	78.22
10	LiMgLg	Barnyard grass	sheath	Libunan, Magdalena	10.178268° N −74.495031° W	light brown	abundant	66.67	90.00	90.00	82.22
11	LMgL	Rice	sheath	Libunan, Magdalena	10.178268° N −74.495031° W	light brown	moderate	35.17	66.33	90.00	63.83
12	BPgL	Rice	sheath	Binan, Pagsanjan	14.271785° N 121.436265° E	light brown	moderate	43.33	77.67	89.17	70.06
13	BPL	Rice	sheath	Buboy, Pagsanjan	14.24° N 121.43° E	light brown	abundant	60.33	90.00	90.00	80.11
14	CPL	Rice	sheath	Cabanbanan, Pagsanjan	14.24694° N 121.430908° E	light brown	moderate	52.67	89.67	90.00	77.44
15	SPL	Rice	sheath	Sabang, Pagsanjan	14.26° N 121.43° E	brown	abundant	55.83	83.33	89.17	76.11
16	LScL	Rice	sheath	Labuin, Santa Cruz	14.254683° N 121.395612° E	light brown	moderate	61.83	90.00	90.00	80.61
17	PScL	Rice	sheath	Patimbao, Santa Cruz	14.266121° N 121.414189° E	light brown	moderate	43.50	86.50	90.00	73.33
18	LPL	Rice	sheath	Labuin, Pila	14.248139° N 121.369887° E	light brown	moderate	37.67	77.33	90.00	68.33
19	ScsPL	Rice	sheath	Santa Clara Sur, Pila	14.226484° N 121.368458° E	light brown	moderate	60.00	90.00	90.00	80.00
20	MVL	Rice	sheath	Masapang, Victoria	14.192465° N 121.337007° E	light brown	moderate	48.00	89.33	90.00	75.78
21	NVL	Rice	sheath	Naninaya, Victoria	14.192465° N 121.337007° E	light brown	moderate	30.00	56.83	86.00	57.61
22	SfVL	Rice	sheath	San Francisco, Victoria	14.215199° N 121.339867° E	whitish brown	scarce	33.83	67.00	90.00	63.61
23	TaVL	Rice	sheath	Tangsa, Victoria	14.202753° N 121.337007° E	light brown	moderate	53.83	86.67	89.17	76.56
24	TuVL	Rice	sheath	Tabuan, Victoria	14.202753° N 121.337007° E	light brown	moderate	50.67	90.00	90.00	76.89
25	BCL	Rice	sheath	Bangyas, Calauan	14.18482° N 121.309839° E	light brown	moderate	49.33	86.67	90.00	75.33
26	MCL	nknown grass	basal stem	Masiit, Calauan	14.163578° N 121.305549° E	yellowish brown	moderate	34.50	69.00	90.00	64.50
27	SiCL	Rice	sheath	San Isidro, Calauan	14.157863° N 121.318419° E	light brown	scarce	35.83	68.5	90.00	64.78
28	MBL	Rice	sheath	Maitim, Bae	14.182059° N 121.275512° E	light brown	moderate	36.00	69.83	90.00	65.28
29	ScNL	Rice	sheath	Santa Clara, Nagcarlan	14.149029° N 121.388467° E	light brown	moderate	37.33	70.33	90.00	65.89
30	KPaL	Rice	sheath	Kwatro, Paete	14.367518° N 121.529954° E	light brown	moderate	37.00	73.17	90.00	66.72
31	QPaLr	Rice	sheath	Quinale, Paete	14.360923° N 121.574104° E	light brown	moderate	68.50	90.00	90.00	82.83
32	SPaL	Rice	sheath	Syete, Paete	14.367518° N 121.529954° E	light brown	moderate	57.33	90.00	90.00	79.11
33	IPngL	Rice	sheath	Isla, Pangil	14.403307° N 121.469898° E	light brown	moderate	32.50	66.17	90.00	62.89
34	NMaL	Rice	sheath	Nanguma, Mabitac	14.444753° N 121.422663° E	light brown	moderate	64.50	90.00	90.00	81.50
35	PMaL	Rice	sheath	Paagahan, Mabitac	14.450122° N 121.405615° E	light brown	moderate	60.00	90.00	90.00	80.00
36	BFL	Rice	sheath	Batuhan, Famy	14.436914° N 121.442761° E	light brown	moderate	30.33	63.83	90.00	61.39
37	D1FL	Rice	sheath	Dunghak 1, Famy	14.473008° N 121.484245° E	light brown	scarce	35.83	66.17	89.67	63.89
38	MFL	Rice	sheath	Mayputat, Famy	14.473008° N 121.484245° E	light brown	moderate	59.83	90.00	90.00	79.94
39	HSL	Rice	sheath	Halayhayin, Siniloan	14.428391° N 121.465614° E	light brown	moderate	33.67	63.67	90.00	62.44
40	MaSL	Rice	sheath	Makatad, Siniloan	14.436155° N 121.482751° E	yellowish brown	moderate	54.83	89.33	90.00	78.06
41	BIG	Mungbean	stem	Pili drive, UPLB	14.163158° N 121.249706° E	yellowish brown	moderate	35.17	65.50	90.00	63.56
42	NCPC	Mungbean	stem	NCPC, UPLB	14.167535° N 121.243282° E	yellowish brown	moderate	29.50	55.67	80.33	55.17

^a^ Observed from 10-day-old cultures. ^b^ Qualitative visual estimate. ^c^ Means of two trials with three replications each.

**Table 2:** Sclerotial and microscopic characteristics of different *Rhizoctonia* isolates collected from rice-producing areas in Laguna, Philippines.

#	Isolate	Color of Sclerotia	Sclerotia Formation Pattern	Mean Sclerotia Diameter (mm) ^a^	Sclerotial Production ^b^	Mean Hyphal Width (µm) ^a^	Nuclear Count	Binucleate (BNR)/ Multinucleate (MNR)	Molecular Identification
1	ALQ	brown/dark brown	scattered	1.69	fair	7.57	5	MNR	*R. solani* AG1-IA
2	SLQ	white/brown	scattered	0.81	excellent	6.90	2	BNR	ND
3	BaMjL	brown/dark brown	scattered	2.48	fair	8.04	5	MNR	*R. solani* AG1-IA
4	BuMjL	brown/dark brown	scattered	1.66	moderate	8.07	9	MNR	*R. solani* AG1-IA
5	IbMjL	brown/dark brown	central	1.68	fair	7.85	8	MNR	*R. solani f. sp. sasakii*
6	TrMjL	white/brown	scattered	0.77	excellent	ND	2	BNR	ND
7	BMgL	salmon	scattered	ND	excellent	7.37	7	MNR	*R. oryzae*
8	CMgL	brown/dark brown	scattered	2.06	good	7.56	6	MNR	*R. solani* AG1-IA
9	IbMgL	salmon	central	ND	excellent	7.26	8	MNR	*R. oryzae*
10	LiMgLg	brown/dark brown	peripheral	1.76	very good	7.05	6	MNR	*R. solani* AG1-IA
11	LMgL	white/brown	scattered	0.63	excellent	7.16	2	BNR	ND
12	BPgL	brown/dark brown	scattered	1.48	moderate	7.48	9	MNR	*R. solani* AG1-IA
13	BPL	brown/dark brown	scattered	1.97	moderate	7.84	10	MNR	*R. solani* AG1-IA
14	CPL	brown/dark brown	scattered	2.69	fair	7.29	6	MNR	*R. solani* AG1-IA
15	SPL	brown/dark brown	scattered	1.83	fair	ND	6	MNR	*R. solani* AG1-IA
16	LScL	NA	NA	NA	poor	9.43	6	MNR	*R. solani* AG1-IA
17	PScL	NA	NA	NA	poor	7.69	7	MNR	*R. solani* AG1-IA
18	LPL	white/brown	scattered	0.86	excellent	ND	2	BNR	ND
19	ScsPL	brown/dark brown	scattered	1.68	fair	7.93	7	MNR	*R. solani* AG1-IA
20	MVL	brown/dark brown	scattered	1.89	moderate	6.12	7	MNR	*R. solani* AG1-IA
21	NVL	white/brown	scattered	0.81	excellent	6.33	2	BNR	*R. oryzae-sativae*
22	SfVL	white/brown	central	0.8	excellent	6.04	2	BNR	ND
23	TaVL	NA	NA	NA	poor	7.31	6	MNR	*R. solani* AG1-IA
24	TuVL	brown/dark brown	peripheral	2.92	good	7.93	6	MNR	*R. solani* AG1-IA
25	BCL	brown/dark brown	scattered	1.21	moderate	7.94	7	MNR	*R. solani* AG1-IA
26	MCL	white to gray	ring	1.39	excellent	5.07	8	MNR	*R. solani* AG1-IA
27	SiCL	white/brown	scattered	0.87	excellent	7.20	2	BNR	ND
28	MBL	white/brown	central	0.71	excellent	6.27	2	BNR	ND
29	ScNL	white/brown	central	0.54	excellent	6.28	2	BNR	*R. oryzae-sativae*
30	KPaL	white/brown	scattered	0.77	excellent	6.45	2	BNR	ND
31	QPaL	brown/dark brown	peripheral	2.52	good	6.96	7	MNR	*R. solani* AG1-IA
32	SPaL	brown/dark brown	scattered	1.52	fair	7.51	7	MNR	*R. solani* AG1-IA
33	IPngL	white to brown	scattered	0.78	excellent	5.82	2	BNR	ND
34	NMaL	brown/dark brown	scattered	2.54	fair	8.08	8	MNR	*R. solani* AG1-IA
35	PMaL	brown/dark brown	scattered	2.05	fair	7.35	6	MNR	*R. solani* AG1-IA
36	BFL	white/brown	scattered	0.64	excellent	ND	2	BNR	ND
37	D1FL	white/brown	scattered	0.61	excellent	6.09	2	BNR	*R. oryzae-sativae*
38	MFL	brown/dark brown	peripheral	1.91	very good	7.28	6	MNR	*R. solani* AG1-IA
39	HSL	white/brown	scattered	0.84	excellent	ND	2	BNR	ND
40	MaSL	salmon	ring	ND	excellent	8.62	10	MNR	*R. oryzae*
41	BIG	NA	NA	NA	poor	5.02	2	BNR	*Ceratobasidium* sp. AG-L
42	NCPC	NA	NA	NA	poor	4.60	2	BNR	*Ceratobasidium* sp. AG-A

**^a^** Average of 20 observations. **^b^** Number of sclerotia/dish: poor (0), fair (1–10), moderate (11–20), good (21–40), very good (41–60), and excellent (>60) [15]. ND, not determined; NA, not applicable because of poor sclerotial production.

**Table 3:** Mega Basic Local Alignment Search Tool (BLAST) search results of the deduced ITS sequences using National Center for Biotechnology Information (NCBI) GenBank. database for species identification.

Isolate	Percent Similarity (%)	Species Identification	Accession No of Standard Isolates	Country of Origin
IbMjL	99	*Rhizoctonia solani f. sp. sasakii*	KF570312.1	India
D1FL	100	*Ceratorhiza oryzae-sativae*	DQ307249.1	China
NVL	97	*Ceratorhiza oryzae-sativae*	FJ667260.1	Japan
ScNL	100	*Ceratorhiza oryzae-sativae*	DQ307249.1	China
LiMgLg	97	*Rhizoctonia solani*	JF701789.1	India
MCLgb	98	*Ceratobasidium* sp. AG-Ba	KF176604.1	China
NCPC	99	*Ceratobasidium* sp. AG-L	FJ515884.1	China
BIG	99	*Ceratobasidium* sp. AG-A	KC782943.1	Italy
BMgL	100	*Waitea circinata*	EF429315.1	China
IbMgL	90	*Waitea circinata*	HM597138.1	USA
MaSL	100	*Waitea circinata*	EF429315.1	China

**Table 4:** Disease severity on rice var. TN1, barnyard grass, tomato var. Athena, and mungbean var. Pagasa 3 with selected *Rhizoctonia* isolates.

Isolate	Rice	Barnyard Grass	Tomato	Mungbean
BPgL	41.85 ^b^	58.78 ^a^	73.00 ^d^	24.08 ^a^
IbMjL	40.54 ^b^	47.23 ^a,b^	62.75 ^d^	25.42 ^a^
QPaL	40.48 ^b^	58.09 ^a^	62.75 ^d^	23.00 ^a^
D1FL	16.72 ^c^	1.66 ^b^	20.50 ^a^	0
NVL	20.45 ^c^	0.27 ^b^	20.50 ^a^	0
ScNL	15.36 ^c^	1.24 ^b^	20.50 ^a^	0
BMgL	32.47 ^b^	18.68 ^a,b^	26.00 ^a,b^	0
IbMgL	39.69 ^b^	25.168 ^a,b^	48.00 ^b,c,d^	0
MaSL	41.24 ^b^	36.44 ^a,b^	31.50 ^a,b,c^	0
LiMgL	51.42 ^a^	49.40 ^a,b^	20.50 ^a^	25.50 ^a^
MCL	ND	1.36 ^b^	37.00 ^a,b,c^	0
NCPC	ND	3.44 ^b^	37.00 ^a,b,c^	ND
BIG	ND	ND	53.50 ^c,d^	ND

Means with the same letter are not significantly different at *p* ≤ 0.05 based on Fisher’s protected least significant differences (LSD). ND, not determined.
